# The Use of Wearable Sensors for the Movement Assessment on Muscle Contraction Sequences in Post-Stroke Patients during Sit-to-Stand

**DOI:** 10.3390/s19030657

**Published:** 2019-02-06

**Authors:** Wei-Chun Hsu, Chao-Chin Chang, Yi-Jia Lin, Fu-Chi Yang, Li-Fong Lin, Kuan-Nien Chou

**Affiliations:** 1 Graduate Institute of Biomedical Engineering, National Taiwan University of Science and Technology, Taipei 10607, Taiwan; D10422205@mail.ntust.edu.tw (C.-C.C.); jiajia527@gmail.com (Y.-J.L.); 2 Graduate Institute of Applied Science and Technology, National Taiwan University of Science and Technology, Taipei 10607, Taiwan; 3 National Defense Medical Center, Taipei 11490, Taiwan; 4 Department of Neurology, Tri-Service General Hospital, National Defense Medical Center, Taipei 11490, Taiwan; fuji-yang@yahoo.com.tw; 5 Department of Physical Medicine and Rehabilitation, Shuang-Ho Hospital, Taipei Medical University, Taipei 11031, Taiwan; 08168@s.tmu.edu.tw; 6 School of Gerontology Health Management, College of Nursing, Taipei Medical University, Taipei 11031, Taiwan; 7 Department of Neurosurgery, Tri-Service General Hospital, Taipei 11490, Taiwan

**Keywords:** wearable sensors, electromyography signal, stroke, sit-to-stand, contraction of the muscles

## Abstract

Electromyography (EMG) sensors have been used to study the sequence of muscle contractions during sit-to-stand (STS) in post-stroke patients. However, the majority of the studies used wired sensors with a limited number of placements. Using the latest improved wearable technology with 16 sensors, the current study was a thorough investigation to evaluate the contraction sequences of eight key muscles on the trunk and bilateral limbs during STS in post-stroke patients, as it became feasible. Multiple wearable sensors for the detection of muscle contraction sequences showed that the post-stroke patients performed STS with abnormal firing sequences, not only in the primary mover on the sagittal plane during raising, but also in the tibialis anterior, which may affect anticipatory postural adjustment in the gluteus medius, which may affect balance control. The abnormal tibialis anterior contraction until the early ascending phase and the delayed firing of the gluteus muscles highlight the importance of whole-kinetic-chain monitoring of contraction sequences using wearable sensors. The findings can be helpful for the design of therapeutic exercises.

## 1. Introduction

Strokes are a global health care problem [1]. The majority of post-stroke patients with disability need help with mobility, self-care, and household activities [2]. Sit-to-stand (STS) is a common daily activity that is essential for upright mobility. Thus, it can significantly affect the functional mobility and quality of life for post-stroke patients. Moreover, it is an important determinant for the independence of post-stroke patients. Studies that explore movement deviation during STS in post-stroke patients often rely on a state-of-the-art motion capture system [2,3,4]. However, these kinds of systems are often expensive and are not available in clinical or household settings. Thus, wearable sensors that allow for the exploration of the reasons for deviations in STS movement would be beneficial for post-stroke patients.

Using traditional motion analysis, temporal parameters while performing a task are essential movement parameters. Post-stroke patients have been reported to raise from a chair with a long STS duration, which has been considered as an indicator of deficits in functional mobility [5,6], and is associated with a higher fall risk [7]. Using force-plates, ground reaction force (GRF) can be obtained, which allows for the observation of the loading muscle group during a task. During STS, post-stroke patients showed asymmetric GRF, revealing a lower magnitude of vertical GRF on the affected side compared with the non-affected side [3,7,8]. The reason for the reported symmetrical weight distribution could be due to bias in perception post-stroke [8] or because of impaired neuromuscular control, which results in inappropriate sequences of muscle contraction.

Electromyography (EMG) has been used to study neuromuscular coordination during STS in young subjects [9,10,11,12,13,14]. Although the sequences of muscle contraction showed high variability as indicated by the large standard errors [11], there was some agreement found in the literature. At the preparation phase of STS, while the buttock has not yet left the chair, the tibialis anterior (TA) should contract to stabilize the foot and to help the body move forward by rotating the shank [11,15,16]. With the movement of the trunk flexion prior to seat-off, the contraction of the rectus abdominis (RA) [14] and the rectus femoris (RF) [11,16] helps with trunk and hip flexion and knee stabilization. It has also been reported that RA is the muscle that first contracts, even earlier than TA [14]. Then, the erector spinea (ES), bicep femoris (BF), and gluteus maximus (GMax) contract to generate movement at the hip to slow down flexion, followed by extension. The vastus lateralis (VL) also contracts to extend the knee [11]. Toward the latter ascending phase, the gastrocnemius (Gas) [11,16], the soleus (SOL), and GMax were the last muscles to be activated [9] for the stabilization of the foot on the floor [11] and for the stabilization of the trunk.

Wearable sensors have also been used to study the sequences of muscle contraction in post-stroke patients [12,17,18,19,20]; however, a majority of the studies had limited numbers of sensor placements, and some of them used wire sensors [12,18], which may increase noise to the signal and may disturb movement [17,19]. Thus, using current improved wearable multi-sensor technology, a thorough investigation of the EMG features, for not only the key muscles in the lower extremities but also the trunk, without disturbing the studied movement, has become feasible. The analysis, including the contraction sequences, for a set of muscle groups in terms of onset, offset, and the occurrence of their peak values, is helpful in the development of clinical training programs for the performance of STS in post-stroke patients. Therefore, the purpose of the current study was to use 16 wearable sensors to monitor and evaluate the contraction sequences of eight key muscles on bilateral limbs, based on the time of onset and offset as well as the occurrence of their peak values during STS in post-stroke patients, and healthy control groups. Group comparisons were performed to investigate the deviated EMG features in post-stroke patients.

## 2. Materials and Methods

### 2.1. Participants

Two groups, including stroke and heathy participants, were recruited in the study. In the stroke group, the participants were six men and four women with hemiplegia secondary to a cerebrovascular accident (mean age, 62.1 ± 7.1 years; mean height, 162.8 ± 5.2 cm; mean weight, 66.7 ± 10.7 kg). The patients were medically stable and presented with no additional peripheral or central nervous system dysfunction in clinical observation, such as cerebellar signs, Parkinsonism, Ménière’s syndrome, or peripheral neuropathy. In the healthy group, the participants were six men and four women (mean age, 62.8 ± 5.5 years; mean height, 166.1 ± 5.2 cm, mean weight, 68.5 ± 13.9 kg, all right-side-limb dominant). None of the participants exhibited symptoms of musculoskeletal disorders or obvious cognitive deficits that would prevent them from following instructions during the experiment. All participants had the ability to perform STS movement independently. Each participant understood and provided informed consent before the experiment. All participants were informed of the study purpose, and signed the consent form approved by the Ethics Committee (Protocol No. 16-079-B1).

### 2.2. Instruments and STS Cycle

**Figure 1:**
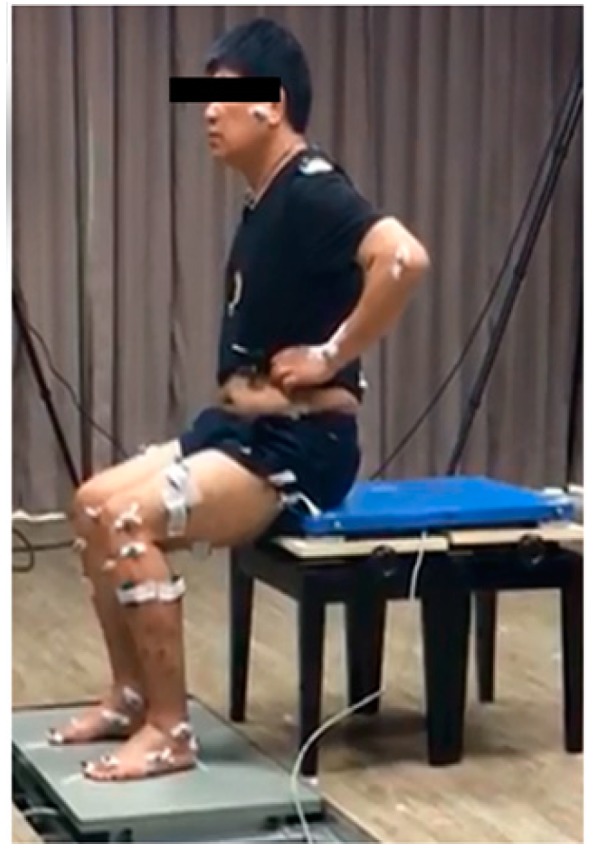
Sensors attached to a participant to detect eight bilateral Electromyography signals.

Sixteen wearable sensors (Delsys Trigno, Natick, MA, USA) (Figure 1), which allow for the detection of EMG signals, were attached to the participant bilaterally to monitor and evaluate the muscle contraction electrical activity and sequences (Figure 1). The EMG sensor signal sampling rate was 2000 samples/s, its bandwidth was 20–450 Hz, the baseline noise was <750 nV, and common-mode rejection ratio (CMRR) was >80 dB. The attachment positions of the EMG sensors were to the ES, RA, GMax, gluteus medius (GMed), BF, RF, SOL, and TA [21]. Infrared retroreflective markers were attached to the bilateral bony landmarks of the thigh and shank, with their trajectories being captured by a motion capture system (Qualysis Oqus 7+, Göteborg, Sweden) with a 200 Hz sampling rate. Two force plates (AMTI OR6-7, Watertown, MA, USA) were placed on the floor under each foot to measure ground reaction force (GRF), and one force plate (Kistler 9260AA, Winterthur, Switzerland) was placed on the chair under the buttocks to measure chair reaction force. The sampling rate of all the three force plates was 2000 Hz. All of the signals from the EMG sensors, the force plates, and the motion capture system were synchronized. Signals from the wearable EMG sensors were used as the main indicator for evaluating the sequences of muscle activation, while data from force plates and the motion capture system served only for defining the movement cycle.

**Figure 2:**
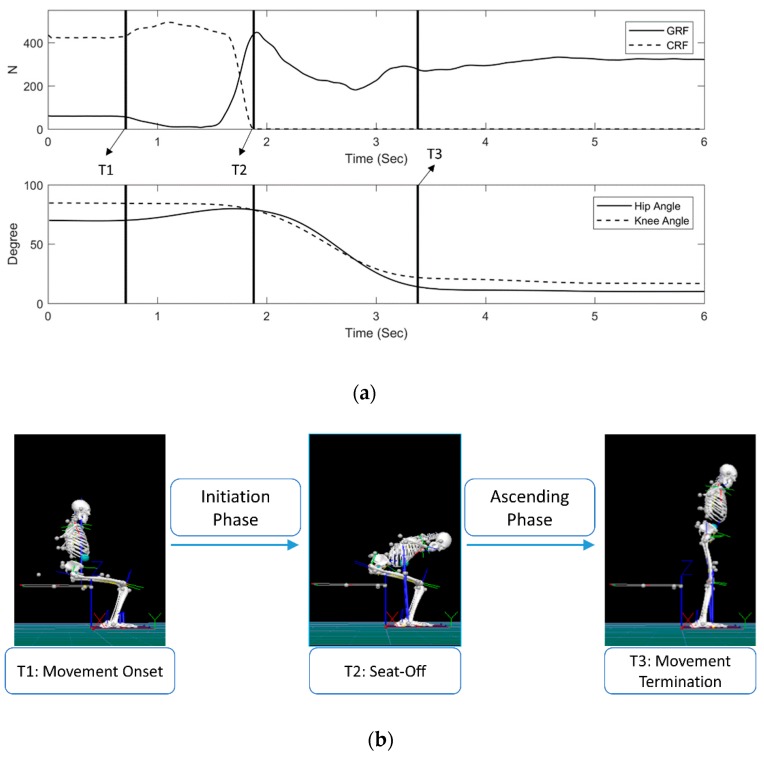
(**a**) T1: The change of the ground reaction force value exceeds the range of the mean value of its baseline with two standard deviations; T2: The vertical chair reaction force from the chair reaches zero; and T3: The hip or knee angle reaches minimum fluctuation; (**b**) the initiation phase was marked from T1 to T2, and the ascending phase from T2 to T3. GRF, ground reaction force. CRF, chair reaction force.

The STS process was divided into initiation and ascending phases by three events. The first event, T1 (onset of STS), was defined as the instant when the change in GRF values exceeded the range of the mean value of its baseline with two standard deviations. The second event, T2 (seat-off), was defined as the time when the vertical chair reaction force from the chair became zero. The third event, T3 (end of STS), was defined as the first frame of static status of standing, in which the hip or knee angle reached minimum fluctuation. For the calculation of the initiation phase, ascending phase, and total duration, the period from T1 to T2 was marked as the initiation phase, the period from T2 to T3 was marked as the ascending phase, and the total duration was from T1 to T3. Figure 2 shows the cycle definition. The movement cycle was used to normalize all of the biomechanical variables and muscle activation times to 100%. The seat-off timing was normalized to determine its percentage during the movement cycle.

### 2.3. Experiments Procedure

Each participant was asked to perform STS at a self-selected pace from a chair that can have its height adjusted. The chair was set to 100% of the knee height of each participant, defined by the height of the fibular head in the sitting posture. During performance of the STS task, participants were allowed to practice several trials and then three trials were recorded.

### 2.4. Data Analysis

A pelvis–leg apparatus was defined as a seven-link model. Each link was defined as an orthogonal coordinate system, with the positive direction of the x-axis anterior, the y-axis superior, and the z-axis right, following the International Society of Biomechanics recommendation [22]. EMG signals were then analyzed. Band-pass filters of 50 and 500 Hz were used to filter raw EMG signals, then a fourth-order low-pass Butterworth filter was used to smooth signals [23]. A rest interval baseline with a time window of 50 ms was set, while the mean and SD values of the signals were analyzed during this time window. The mean of the baseline value plus two SD was defined as a threshold. During a time interval greater than or equal to 100 ms, the onset of activation for each muscle was identified as when the EMG signal exceeded the threshold [17]. Relative to seat-off (T2), the mean occurrences of muscle onset and peak time were calculated, and the time before seat-off was set to negative and after seat-off was set to positive. To investigate the distribution of EMG data and to avoid effects from extreme outliers, a boxplot was used to graphically illustrate the onset and peak times for each of the muscles on both sides of stroke and healthy participants. The boxplot was used to comprise the maximum and minimum values in the dataset, as well as the right hinge (third quartile), median, and left hinge (first quartile).

### 2.5. Statistical Analysis

Demographic data of the participants were recorded, including age, height, and weight. An independent t-test was used to examine the differences in the demographic data between the stroke and healthy control groups, including age, weight, and height. The t-test was used to test the null hypothesis that there was no significant difference between the stroke and healthy control groups in age, weight, and height. For all of the extracted variables, the Kolgomorov–Smirnov test was performed to check for a normal distribution. As the assumption of normality was not met for most of the outcome measures, the Mann–Whitney U test was used to examine the group effects (i.e., stroke and healthy controls). The significance level was set at α = 0.05. SPSS version 17 was used for all statistical analyses.

## 3. Results

No significant difference between groups was identified for the temporal parameters (Table 1). Although the difference in duration variables did not reach significant levels, the averaged duration in the stroke group was greater than that required in the healthy group for the initiation phase, ascending phase, and total duration, indicating a trend of longer time required for post-stroke patients to stand up.

The mean onset time of muscle activity is shown in Table 2. Regarding the onset times, significantly delayed onsets of TA, ES, BF, and GMax were found in the unaffected limbs of the stroke group, compared with the dominant limbs of healthy controls. The GMax and GMed were also found to have significantly delayed onset times in the affected limbs of the stroke group, compared with the non-dominant limbs of healthy controls (Table 2).

The mean peak time of muscle activity is shown in Table 3. Regarding the peak times, significantly delayed peak times of TA, RA, BF, GMax, and SOL were found in the unaffected limbs of the stroke group, compared with the dominant limbs of healthy controls. The TA, GMax, and GMed were also found to have significantly delayed peak times in the affected limbs of the stroke group, compared with the non-dominant side of healthy controls (Table 3).

The mean offset time of muscle activity is shown in Table 4. Regarding the offset times, significantly delayed offsets of RA, BF, GMax, and SOL were found in the unaffected limbs of the stroke group, compared with the dominant limb of healthy controls. The GMax and GMed were also found to have significant offsets in the affected limbs of the stroke group, compared with the non-dominant side of healthy controls (Table 4).

## 4. Discussion

Wearable sensors are increasingly used in studies of human motion analysis, through which the unobtrusiveness and light-weight EMG sensors help to investigate muscular activities that produce movement. With a well-defined movement cycle, which allowed for a detailed understanding of the muscle contraction sequences corresponding to the different tasks along the course of the STS cycle, the current study used 16 wearable sensors to monitor and investigate the contraction sequence of eight key muscles on bilateral limbs during STS in post-stroke patients and a healthy control group.

Comparison of the movement duration of STS phases and cycle duration between groups showed no significant differences (Table 1), although there was a trend of longer STS times required for the patients with stroke, which may result in physical limitation among post-stroke patients [5,6], and may be associated with a greater risk of falling [7]. With a slightly longer STS duration, the post-stroke patients performed STS with a different sequence of muscle contractions in terms of onset, offset, and peak time (Table 2, Table 3 and Table 4).

Apart from those that have been documented in young subjects, wearable sensors have also been widely used to study the sequence of muscle contractions in various kinds of populations, including the elderly [9,13,24]. Unlike what has been found in young adults, the delay of the anticipatory contraction of the TA was found in the elderly, suggesting decreased motor control ability [25]. This lack of anticipatory contraction of the TA was even more prominent in our stroke group compared with the healthy control group, revealed by the delayed onset of TA anticipatory contraction (Table 2). In our healthy controls, it seemed that TA still played an important role in anticipatory postural adjustment (APA), because the onset, peak, and offset of their activation were all prior to the beginning of rising, with an activation timing of around 750 ms for the APA phase. In patients with stroke, the ability of the central nervous system to adjust to anticipatory activation of muscles was impaired. Note that the elderly deactivated their TA before the seat-off, while the stroke patients showed persistent TA contraction until the early ascending phase (Table 4). With similar onset timings of SOL, delayed onset and offset of the TA in the stroke group would increase the SOL/TA co-contraction. Previous studies have considered that the co-contraction of SOL and TA is needed for maintaining/increasing balance [13], foot and ankle stability [17,24], and the forward movement of the trunk over the lower limbs [17]. Because SOL was one of the most-affected muscles with the presence of spasticity, there were possible alternations in motor control mechanisms of the ankle inherent to post-stroke patients [19]. Meanwhile, excessive early onset of the SOL should be avoided, because the SOL contracted earlier than the TA and quadriceps in post-stroke fallers, and thus this abnormal activation sequence can be considered a risk factor of fall [18]. Delayed onset of TA and early activation of the SOL during STS have contributed to our understanding of anticipatory and reactional muscular adjustments [17,18], but cannot determine the sequence of activation in multiple locations of key muscle groups that may reveal abnormal muscular activities in post-stroke patients. In the previous studies that investigated the muscular activities during STS in post-stroke patients [17,18], the sensors used were not wireless, so only a few locations had sensors attached, which limited the number of muscles to be examined. With advanced wearable technology, muscular activities can be investigated in more detail.

During the post-stroke recovery, non-use syndrome develops, especially on the affected limb [12,19]. This disuse phenomenon was evident in the GMax and GMed of the affected limbs, which showed a significantly delayed onset (Table 2). During rehabilitation, patients’ attention should be directed to the affected limb. In the unaffected limb, the timing of contraction of RF and GMed in post-stroke patients were found to be similar to those found in the elderly. However, a longer duration of contraction was found in the RA of the unaffected side and prolonged activation was found in the RA, BF, GMax, and SOL, revealed by their significantly later offset times (Table 4). These findings are in agreement with previous studies that indicated that post-stroke patients favor the unaffected limb to stand up [12]. Rehabilitation efforts should also target reducing the use of the unaffected limb for more symmetrical muscle activity of the lower limbs. Moreover, the longer periods of contraction time compared with the healthy group could also be a function of the increased duration of both the initiation and ascending phases (Table 1), which is in agreement with previous findings that indicated that subjects with hemiplegia generated an increased amount of muscle activity over a longer period of time, with greater reliance on the unaffected limb [12].

The abnormal muscle activation reported in this manuscript can be used to highlight the key points of STS training, which aims to enhance the neuromuscular control of the task. The methods established in the current study, which reported the muscle activation sequences across not only the lower limb but also the trunk, could be a good reference for further development of devices that offer warming and/or feedback when abnormal muscular sequences occur during STS. To help in rehabilitation activities, the system can be simplified by replacing the force plates by pressure sensors and by placing a simple accelerometer to define the initiation and termination of the movement.

## 5. Conclusions

With multiple wearable sensors, a thorough investigation of the sequence of muscle activation for muscles in the lower extremities and the trunk was conducted. Using 16 wearable sensors to quantify the time of onset and offset, as well as the occurrence of peak values during STS in post-stroke patients versus healthy controls groups, indicated that the post-stroke patients persistently contracted TA until the early ascending phase, which should be deactivated before seat-off. With similar onset timings of SOL, delayed onsets and offsets of the TA could increase the SOL/TA co-contraction. A significantly delayed onset of GMax and GMed of the affected limbs suggested that attention should be paid not just to the extensor, but also to the stabilizer, for the facilitation and strengthening of muscles on the proximal part around the pelvis and hips to prevent the disuse phenomenon. A thorough understanding of the deviated sequences of muscle contraction is helpful for the development of clinical training programs for the improved performance of STS in post-stroke patients.

## Figures and Tables

**Table 1:** Durations of sit-to-stand (STS) movement and its sub-phases in Stroke and Healthy subjects.

	Stroke	Healthy Controls	Mean Difference of 95% CI	*p*
Initiation phase (s)	0.987 ± 0.326	0.752 ± 0.161	0–0.286	0.100
Ascending phase (s)	1.329 ± 0.443	1.169 ± 0.343	0.096–0.704	0.400
Total duration (s)	2.315 ± 0.692	1.921 ± 0.457	0.016–0.584	0.300
Seat-off (% STS Cycle)	41.665 ± 7.557	38.985 ± 6.224	0.096–0.704	0.400

Values are expressed as means ± SD.

**Table 2:** Onset times of muscular activities in Stroke and Healthy control groups.

Onset Timing of Muscular Activities ^1^ (% STS Cycle) Before Seat-Off (−)/After Seat-Off (+)		Stroke Group	Healthy Control Group	Mean Difference of 95% CI	*p* *
TA	Unaffected/Dominant	−36.571 ± 18.147	−46.959 ± 8.909	0–0.259	0.000 *
	Affected/Non-Dominant	−36.855 ± 16.615	−48.228 ± 12.007	0–0.286	0.100
ES	Unaffected/Dominant	−36.825 ± 10.245	−45.091 ± 7.384	0–0.259	0.000 *
	Affected/Non-Dominant	−39.344 ± 6.748	−44.710 ± 7.665	0–0.286	0.100
RF	Unaffected/Dominant	−40.736 ± 7.973	−39.837 ± 9.766	0.552–1	0.800
	Affected/Non-Dominant	−37.271 ± 10.834	−41.581 ± 7.506	0.416–0.984	0.700
RA	Unaffected/Dominant	−35.642 ± 8.435	−41.137 ± 11.190	0–0.286	0.100
	Affected/Non-Dominant	−34.486 ± 10.646	−39.950 ± 10.531	0–0.286	0.100
BF	Unaffected/Dominant	−28.224 ± 12.302	−39.766 ± 12.785	0–0.259	0.000 *
	Affected/Non-Dominant	−33.951 ± 7.603	−40.399 ± 11.814	0–0.286	0.100
GMax	Unaffected/Dominant	−17.489 ± 16.429	−34.260 ± 8.927	0–0.259	0.000 *
	Affected/Non-Dominant	−19.866 ± 14.707	−37.987 ± 10.070	0–0.259	0.000 *
GMed	Unaffected/Dominant	−25.665 ± 12.841	−24.917 ± 18.036	0.552–1	0.800
	Affected/Non-Dominant	−18.894 ± 19.396	−35.185 ± 12.176	0–0.259	0.000 *
SOL	Unaffected/Dominant	−13.593 ± 17.658	−27.577 ± 19.338	0–0.286	0.100
	Affected/Non-Dominant	−14.336 ± 16.630	−9.497 ± 18.697	0.096–0.704	0.400

^1^ Values are expressed as means ± SD. * *p* < 0.05 indicates a difference between Stroke and Healthy STS groups. TA, tibialis anterior; ES, erector spinea; RF, rectus femoris; RA, rectus abdominis; BF, bicep femoris; GMax, gluteus maximus; GMed, gluteus medius; SOL, soleus.

**Table 3:** Peak times of muscular activities in Stroke and Healthy control groups.

Peak Timing of Muscular Activities ^1^ (% STS Cycle) Before Seat-Off (−)/After Seat-Off (+)		Stroke Group	Healthy Control Group	Mean Difference of 95% CI	*p* *
TA	Unaffected/Dominant	−18.703 ± 12.773	−31.723 ± 8.477	0–0.259	0.000 *
	Affected/Non-Dominant	−17.707 ± 11.124	−30.025 ± 10.165	0–0.259	0.000 *
ES	Unaffected/Dominant	−23.712 ± 10.698	−30.430 ± 9.601	0–0.286	0.100
	Affected/Non-Dominant	−24.703 ± 6.502	−25.734 ± 9.644	0.741–1	1.000
RF	Unaffected/Dominant	−24.055 ± 5.505	−21.546 ± 13.073	0.741–1	1.000
	Affected/Non-Dominant	−18.447 ± 12.602	−15.072 ± 18.552	0.741–1	1.000
RA	Unaffected/Dominant	−10.115 ± 13.512	−21.668 ± 13.368	0–0.259	0.000 *
	Affected/Non-Dominant	−7.560 ± 15.244	−10.189 ± 24.503	0.296–0.904	0.600
BF	Unaffected/Dominant	10.844 ± 17.547	−14.749 ± 17.668	0–0.259	0.000 *
	Affected/Non-Dominant	6.582 ± 25.901	−9.926 ± 24.706	0–0.286	0.100
GMax	Unaffected/Dominant	6.005 ± 14.387	−13.505 ± 20.714	0–0.259	0.000 *
	Affected/Non-Dominant	8.654 ± 15.792	−11.925 ± 10.091	0–0.259	0.000 *
GMed	Unaffected/Dominant	9.535 ± 15.634	−0.905 ± 20.152	0–0.286	0.100
	Affected/Non-Dominant	12.330 ± 17.590	−7.491 ± 16.485	0–0.259	0.000 *
SOL	Unaffected/Dominant	1.396 ± 13.690	−16.481 ± 18.323	0–0.259	0.000 *
	Affected/Non-Dominant	7.390 ± 14.144	7.495 ± 16.692	0.714–1	0.900

^1^ Values are expressed as means ± SD. * *p* < 0.05 indicates a difference between Stroke and Healthy STS groups.

**Table 4:** Offset times of muscular activities in Stroke and Healthy control groups.

Offset Timing of Muscular Activities ^1^ (% STS Cycle) Before Seat-Off (−)/After Seat-Off (+)		Stroke Group	Healthy Control Group	Mean Difference of 95% CI	*p* ^*^
TA	Unaffected/Dominant	1.349 ± 13.577	−5.163 ± 11.901	0.096–0.704	0.400
	Affected/Non-Dominant	0.873 ± 19.117	−10.364 ± 11.62	0–0.286	0.100
ES	Unaffected/Dominant	21.354 ± 18.684	17.126 ± 12.246	0–0.286	0.100
	Affected/Non-Dominant	29.609 ± 12.627	17.327 ± 24.381	0–0.286	0.100
RF	Unaffected/Dominant	26.588 ± 12.493	23.056 ± 17.839	0.096–0.704	0.400
	Affected/Non-Dominant	25.067 ± 13.036	28.773 ± 11.567	0.416–0.984	0.700
RA	Unaffected/Dominant	33.775 ± 12.725	18.548 ± 22.487	0–0.259	0.000 *
	Affected/Non-Dominant	29.205 ± 18.689	16.927 ± 24.429	0–0.286	0.100
BF	Unaffected/Dominant	34.419 ± 18.714	20.265 ± 13.054	0–0.259	0.000 *
	Affected/Non-Dominant	27.457 ± 16.333	21.007 ± 20.719	0.096–0.704	0.400
GMax	Unaffected/Dominant	26.952 ± 18.733	8.986 ± 27.998	0–0.259	0.000 *
	Affected/Non-Dominant	33.920 ± 9.780	15.178 ± 16.764	0–0.259	0.000 *
GMed	Unaffected/Dominant	30.671 ± 17.243	27.750 ± 10.986	0.296–0.904	0.600
	Affected/Non-Dominant	35.596 ± 14.294	13.07 ± 25.459	0–0.259	0.000 *
SOL	Unaffected/Dominant	16.286 ± 13.909	1.078 ± 21.193	0–0.259	0.000 *
	Affected/Non-Dominant	20.803 ± 17.615	18.199 ± 13.802	0.096–0.704	0.400

^1^ Values are expressed as means ± SD. * *p* < 0.05 indicates a significant difference between Stroke and Healthy STS groups.
